# Application of High-Intensity Ultrasound to Improve Food Processing Efficiency: A Review

**DOI:** 10.3390/foods11010122

**Published:** 2022-01-04

**Authors:** Prasad Chavan, Pallavi Sharma, Sajeev Rattan Sharma, Tarsem Chand Mittal, Amit K. Jaiswal

**Affiliations:** 1 Department of Food Technology and Nutrition, Lovely Professional University, Phagwara 144402, India; erprasad.chavan@gmail.com; 2 Department of Processing & Food Engineering, Punjab Agricultural University, Ludhiana 141004, India; psharma9136@yahoo.com (P.S.); sajeevrattan@pau.edu (S.R.S.); tarsem6972@pau.edu (T.C.M.); 3 School of Food Science and Environmental Health, Faculty of Science, Technological University Dublin—City Campus, Central Quad, Grangegorman, D07 ADY7 Dublin, Ireland; 4 Environmental Sustainability and Health Institute (ESHI), Technological University Dublin—City Campus, Grangegorman, D07 H6K8 Dublin, Ireland

**Keywords:** cavitation, ultrasound, food processing, ultrasound-assisted extraction, ultrasound-assisted freezing

## Abstract

The use of non-thermal processing technologies has grown in response to an ever-increasing demand for high-quality, convenient meals with natural taste and flavour that are free of chemical additions and preservatives. Food processing plays a crucial role in addressing food security issues by reducing loss and controlling spoilage. Among the several non-thermal processing methods, ultrasound technology has shown to be very beneficial. Ultrasound processing, whether used alone or in combination with other methods, improves food quality significantly and is thus considered beneficial. Cutting, freezing, drying, homogenization, foaming and defoaming, filtration, emulsification, and extraction are just a few of the applications for ultrasound in the food business. Ultrasounds can be used to destroy germs and inactivate enzymes without affecting the quality of the food. As a result, ultrasonography is being hailed as a game-changing processing technique for reducing organoleptic and nutritional waste. This review intends to investigate the underlying principles of ultrasonic generation and to improve understanding of their applications in food processing to make ultrasonic generation a safe, viable, and innovative food processing technology, as well as investigate the technology’s benefits and downsides. The breadth of ultrasound’s application in the industry has also been examined. This will also help researchers and the food sector develop more efficient strategies for frequency-controlled power ultrasound in food processing applications.

## 1. Introduction

Food is a complex material of water minerals, vitamins, enzymes, proteins, carbohydrates, fats, and other organic ingredients with differing compositions. Global food wastage is recognized as a major challenge for sustainable development as, according to the Food and Agriculture Organization (FAO), almost 17% of all food produced is wasted every year, and the amount is estimated to be 931 million tonnes per year. Along with increasing food production, food preservation, processing, and value addition are vital options taken into consideration to meet the food demand of the rising population. Thermal processing is one of the most common preservation techniques. However, many food ingredients are heat sensitive and can be lost during heat processing. Additionally, rising consumer awareness has increased demands for fresh, higher quality, and microbiologically safer and stable foods and has promoted research on non-thermal methods of food preservation [1,2,3].

Ultrasound, as a non-thermal food processing technology, is applied to bring positive effects in food processing, such as food preservation, improvement in mass transfer, the assistance of thermal treatments, the alteration of texture, and food analysis [4]. Ultrasonic waves (also called supersonic waves) are sound waves in the frequency ranges of 20 kHz to 100 kHz [5]. Ultrasound produces ‘cavitation’ in liquids, pressure variations in gas media, and liquid movement in solid media, respectively [6]. It can be viewed as a form of high-frequency vibration that generates fluid mixing and shear forces on a micro scale [7]. This review aims to explore the fundamental principles of ultrasound generation, understand the cavitation mechanism and recent applications of high-intensity ultrasound in food processing, and understand the pros and cons of ultrasound processing so that it can be utilized as an innovative food processing technology.

Ultrasound covers almost all the unit operations that food is subjected to, ranging from sorting, grading, and preservation to processing and storage. One of the first applications of ultrasound reported in the literature was to degrade the biological polymer. Later on, the applications of ultrasonic cavitation have increased in popularity, such as for the enhancement of chemical reactions, the emulsification of oils, the degradation of chemical and biological pollutants, the inactivation of microorganisms, etc. [8,9]. Although a number of studies concerning the application of ultrasound in food processing are available in the literature, this review intends to comprehensively describe the principles of ultrasound, namely, its opportunities and advances in its application in the food industry. Moreover, we cover a range of processing operations where ultrasound has been used in the present day to enhance the processing of foods.

## 2. Generation of Ultrasound

The piezoelectric effect was discovered by the Curie brothers in 1880. Later, French researcher Langevin was the pioneer of ultrasound who described the principle of the generation of ultrasonic waves as based on the piezoelectric effect in 1917. Ultrasound is generated by electrical energy supplied to a piezoelectric material referred to as the transducer, in which the piezoelectric materials convert electrical energy into mechanical vibrations of a particular frequency [4]. When mechanical pressure is applied to two opposite faces of a crystal, an equal and opposite electric charge is developed on the other faces, resulting in a potential difference. Conversely, when the opposite faces of the crystal are brought under potential difference, a change in the dimension (a mechanical contraction or expansion) in the other faces would occur according to the direction of the applied potential difference [1]. This phenomenon is called the piezoelectric effect. Quartz, Tourmaline, and Roche salt are examples in which the piezoelectric effect has been observed.

Ultrasonic wave-producing systems consist of a generator, a transducer, and an application system. A generator produces mechanical or electrical energy while a transducer converts this energy into sound energy at ultrasonic frequencies [10].

## 3. Classification of Ultrasound Applications

**Figure 1:**
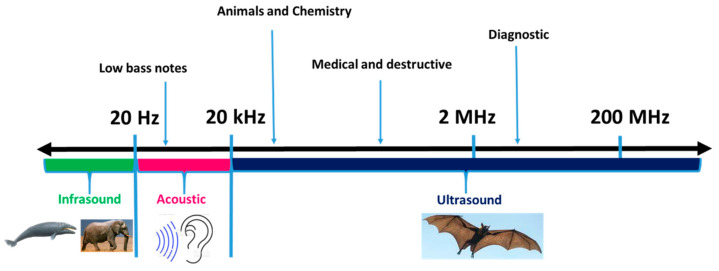
Classification of Ultrasound.

Ultrasound is known as a green novel technology due to its role in environmental sustainability. Ultrasound waves are classified into four different categories based on the mode of vibration of the particles in the medium, with respect to the direction of propagation of the wave, viz., longitudinal waves, transverse waves, surface waves, and plate waves [11]. Depending upon the frequency of the sound, ultrasound waves are divided into three categories, as shown in Figure 1: viz., Power Ultrasound (20–100 kHz), High-Frequency Ultrasound (100 kHz–1 MHz), and Diagnostic Ultrasound (1–500 MHz). Ultrasonic waves of frequency 20–100 kHz are used in chemical systems. Waves of frequency 1–10 MHz are used in animal navigation and communication, for the detection of cracks in solids, as well as for diagnostic purposes [7,10,12].

The application of ultrasound in the food industry is divided into low energy and high energy approaches depending upon the sound power (W), sound intensity (W/m^2^), or sound energy density (Ws/m^3^) used. An ultrasound frequency higher than 100 kHz and intensity below 1 W/cm^2^ is preferred for low-energy applications that normally do not change the physical or chemical properties of the material through which they propagate [10]. These are normally used for analytical applications, such as the determination of the physico-chemical properties of the materials, composition, ripeness, firmness, sugar content, and acidity of fruits and vegetables [13]. Conversely, high-energy (power ultrasound) applications use frequencies between 20 and 100 kHz, and intensities that are higher, in the range of 10–1000 W/cm^2^ can alter the physicochemical properties or structure of a material [14]. Today, it can be more effectively used for enzyme inactivation, the enhancement of drying and freezing processes, the extraction of essential oils, the control of crystallization processes, and the degassing of liquid foods [13,15,16].

Ultrasound techniques can be used in conjunction with other treatments, such as pressure, temperature, or pressure and temperature together, to increase the overall process efficiency. Ultrasonication is the application of ultrasound itself at low temperatures to heat-sensitive products. When ultrasound is used in combination with heat, it is sonication, which inactivates microorganisms more effectively than heat alone. Manosonication is the process in which ultrasound is applied in combination with pressure, which has a higher inactivation efficiency than ultrasound alone at the same temperature. Manothermosonication is the combined method of heat, ultrasound, and pressure that maximizes the cavitation implosion in the media and increases the level of inactivation [17,18].

## 4. Cavitation

Ultrasonic waves, like other sound waves, pass through any physical medium, compressing and stretching the molecular spacing of the medium through which they pass. During the negative pressure cycle, the liquid is pulled apart at sites containing a gaseous impurity, known as a ‘weak spot’ in the liquid [19]. When the negative pressure is large enough, the distance between the molecules of the liquid exceeds the maximum molecular distance required to hold the liquid in contact, and then the liquid breaks down, creating voids. These voids are called ‘cavitations’. This negative pressure results in the formation of gas bubbles, which grow continuously by a rectified diffusion mechanism characterised by the net flow of gas from the liquid to bubbles [20].

Cavitation liquids can contain thousands of such voids, and the size of the voids can be roughly estimated by Equation (1):

F × R = 3,(1)

where F is the frequency in MHz and R is the radius in microns. Linear resonance radius is a more accurate form of the equation, which can be calculated using Equation (2):

Rr = √(3γP∞/ρω2).(2)

where, Rr is linear resonance radius, γ is the specific heat ratio of the gas inside the bubble, P∞ is the ambient liquid pressure, ρ is the liquid density and ω is the angular frequency of ultrasound.

The bubble formed by this process may then grow until it reaches a critical size, known as its ‘resonance size’. which depends upon the applied frequency of the sound wave. As the liquid compresses and stretches, the cavitations can behave in two ways. The bubbles formed at fairly low ultrasonic intensities (1–3 W/cm^2^) are termed “stable cavitations”. “Transition cavitations” are formed using acoustic intensities of more than 10 W/cm^2^. Transition bubbles expand to a radius of at least twice their initial size, before collapsing violently on the compression cycle. It is estimated that a temperature of 4500–5000 K and pressure > 1000 atm can be generated by the collapsing of each bubble [21]. The temperature and pressure changes resulting from these explosions are the main bactericidal effect that kills the bacteria, but they are very localized [22].

### 4.1. Factors Affecting the Cavitation

#### 4.1.1. Frequency

The size of the bubbles and, consequently, the strength of the implosion are determined by frequency [23]. At higher frequencies, the compression and rarefaction caused by the sonic wave are short enough to separate liquid molecules; thus, no cavitation is obtained. Low-frequency ultrasound generates larger bubbles, which on violent collapse can generate higher localised temperatures and pressures. To achieve cavitations at higher frequencies, the intensity of the applied sound must be increased to overcome the cohesive force of the liquid media to form voids [24].

#### 4.1.2. Intensity

A direct relation exists between the intensity of sonication and the amplitude of the vibration of the ultrasonic source [24]. Higher-amplitude vibrations should be employed when working with samples of higher viscosity. High amplitudes lead to high sonication intensities and high sonication effects promote some desired effects.

#### 4.1.3. State of the Material

In the food sector, using ultrasound in a liquid medium is the simplest and most prevalent procedure. When ultrasound is administered to a liquid, the main phenomenon responsible for the consequences is cavitation. Cavitation happens when the microbubbles in the liquid grow in size as a result of the ultrasonic waves’ cycles of high and low pressure until they become unstable and burst, releasing a huge quantity of energy (theoretically, up to 5000 K and 1000 atm) [25]. When an ultrasonic wave passes through a solid medium, the ‘sponge effect’ occurs due to alternating contractions and expansions that aid in the transfer of the matter to the medium around the solid. Furthermore, mechanical stress can lead to the creation of microchannels in the solid’s interior, facilitating mass transfer activities. The cavitation event is unlikely to occur in the liquid phase of the solid matrix in this scenario [26].

#### 4.1.4. Temperature, Pressure, and Viscosity

Temperature and static pressure are important components in cavitation conditioning, and they can be changed depending on the application. As a result, as the temperature rises, the viscosity of the medium decreases and the vapour pressure rises, allowing bubbles to develop. However, as the temperature rises, the volume of the vapour inside the bubbles grows, cushioning the collapse and reducing cavitation intensity. As a result, it is thought that there is a temperature at which acoustic cavitation is greatest [27]. When pressure rises, cavitation is hampered, but when the implosion occurs, the energy released is far greater. When it comes to the viscosity of the medium, bubble production becomes more difficult as the viscosity increases, yet the implosion becomes stronger [26].

## 5. Applications of Ultrasonic Waves in Food Processing

### 5.1. Microbial Inactivation

Thermal treatments such as pasteurization are mostly considered useful for bacterial inactivation, but undesirable effects such as unwanted flavours and nutrient losses have encouraged research on novel processing techniques. Ultrasound treatment is applied as a processing aid to inactivate microbes. Various textural and physiological changes, i.e., the thinning of cell membranes and the production of free radicals, are the main mechanisms by which microorganism inactivation takes place [28]. Transient cavitation will produce localized hot spots up to 4500–5000 K, and pressures > 199 MPa produce shock waves and free radicals, whereas stable cavitation will produce micro streaming accompanied by high shear [29]. All these contribute to damage of the cell wall and membrane, resulting in cell death. It was reported that ruptured and disintegrated cells cannot be reviewed, which is advantageous over some other techniques in which damaged cells can recover if they encounter the right environmental conditions (temperature, pH, water activity, and nutrients) [29].

The resistance offered by five groups of microorganisms to the ultrasonic inactivation is in the order of spores > fungi > yeast > gram-positive cells > gram-negative cells. The resistance of viruses to ultrasound is high, but not enough data is yet available to compare it with the other microorganisms. Microbial destruction can also be accomplished by combining ultrasound with other treatments, such as heat (thermosonication), low static pressure (monosonication), ultraviolet light, or antimicrobials. Sonication combined with high pressures and temperatures (manothermosonication at 400 kPa/59 °C) was applied by Lee et al. [30] for the control of *Escherichia coli (E. coli) K12* populations in apple cider, and they achieved a 5-log reduction in 1.4 min and in 3.7 min when sonication was combined with a lethal temperature—whereas treatment via sonication alone takes 15.9 min for the same *E. Coli* reduction. A sonication of yeast cells in *Saccharomyces cerevisiae* 2200 strain using a 20-kHz horn-type sonicator was carried out by Fabiszewska [31]. After sonication, the count of live yeast cells decreases by 100 to 1000 times, compared to their initial count, expressed as Colony Forming Units CFU/cm^3^. The efficacy of microbial destruction is governed by amplitude and frequency of the ultrasonic waves, the exposure/contact time, the composition and volume of the food to be processed, and the conditions [28]. It was observed that the number of bubbles undergoing cavitation per unit of time increases at higher amplitudes, which resulted in a higher inactivation rate of the microorganisms. The majority of the research has been conducted to determine the effect of ultrasound on the microbial inactivation of fruits and vegetables [32,33,34,35]. The inactivation of microorganisms using heat treatments and ultrasound and their D values (time for 90% reduction of microorganisms) are summarized in Table 1.

### 5.2. Ultrasonic Cutting

**Figure 2:**
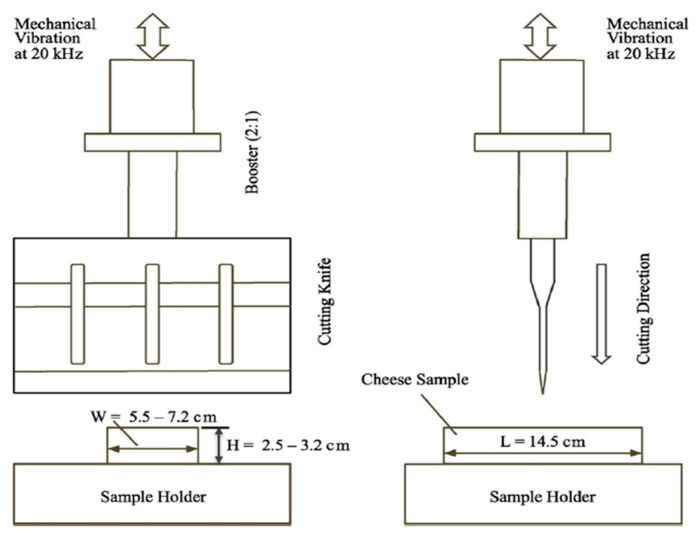
Ultrasonic cutting system with cutting knife (reprinted with permission from Ref. [43] Copyright 2016 Elsevier).

Because of the increasing demand for an improved quality of food-cutting processes with high accuracy, excellent cut faces, reduced smearing, low product loss, less deformation, less tendency to shatter for brittle products, and the ability to handle sticky or brittle foods, ultrasonic cutting is becoming increasingly important [6,42]. Ultrasonic cutting is the size-reduction operation that utilizes the vibrating energy of the ultrasound, which superposes with the conventional blade movement, to improve the cutting efficiency and quality of the product [43]. An ultrasonic cutting machine is composed of an ultrasonic transducer, a power supply unit, a horn, and a cutting knife, as shown in Figure 2. In the ultrasonic cutting mechanism, due to the high-frequency vibrations of the cutting blades, the food and cutter experience alternative contact and separation at a high deformation rate, though smaller deformations result in a reduced total cutting force and an avoidance of transversal cracks and crumbling, with a reduction of cutting-surface roughness [42].

The piezo-electric transducer converts the voltage that is supplied by a generator into a corresponding mechanical displacement at the outlet of the piezo element. The amplifier then transmits this displacement to the sonotrode, which induces an oscillation of a defined frequency at the cutting edge of the sonotrode [36]. The cutting mechanism is more effectively used for cutting viscoelastic and viscoplastic foods, fragile and frozen foods, as well as heterogeneous products. The quality of food cut by ultrasonics, and the cutting efficiency, depends on the geometry of the cutter, the direction of the vibration of the cutter relative to the cutting direction, the frequency and amplitude of the cutter, and the product’s properties, such as its microstructure, moisture or fat content, or temperature sensitivity [6,42,44].

Ultrasonic cutting technology has been predominantly applied for cheese, bakery and confectionery products, and foods with smooth textures. Ultrasonically cut food showed a shining and smooth surface appearance of samples as compared to control [43]. The technique is most suitable for porous products that possess high compressibility, high elasticity, low adhesiveness, and have a low content of lubricant liquid, which leads to frictionless cutting and deformation forces along the flanks of the cutting blade [45]. Zahn et al. [46] studied the relationship between cutting force requirement and magnitude and cutting velocity for baked materials (white bread, malted bread, sunflower grain bread, hamburger buns, milk rolls, and pound cakes) [43]. It was observed that cutting work (Wc) decreases with an increase in vibration speed. However, a combination of the highest cutting velocity and the maximum vibration speed resulted in a significant reduction of cutting work for white bread. An inverse relationship exists between the average cutting force and the contact time between the product and the cutting device. For various foods, an ultrasonic frequency or amplitude (or both) is inversely proportional to cutting forces, whereas, irrespective of ultrasonic assistance, linear cutting velocity is directly proportional to the resulting forces. Additionally, as the cutting depth increases, the power consumption of the cutting system increases the amount of work required for ultrasound cutting (WUS), which is directly proportional to protein content and inversely proportional to the fat dry matter and moisture to the solid-non-fat ratio. Thus, reduced product deformation, higher cutting quality, and lower amount of product waste make the ultrasound cutting technique more impressive [45].

### 5.3. Ultrasonic Drying

Drying of the food materials is the most common and promising method for stabilizing the product. Traditional drying techniques cause adverse effects, such as shrinkage, discolouration, and the oxidation of vitamins. Additionally, rising energy costs, increasing quality requirements at reduced nutritional losses, and adverse environmental impacts have resulted in an increased interest in the development of modern food-drying technologies [47,48]. The use of ultrasound accelerates the drying process without causing a severe temperature change. It has been suggested that mass transport kinetics can be increased by using high-intensity ultrasound. Moreover, ultrasound can decrease the boundary layer thus resulting in a decrease in resistance to mass transfer required for the drying process [49]. An experimental study was carried out by Ortuno et al. [50] on the convective drying kinetics of orange peel slabs at 40 °C and 1 m/s with and without power ultrasound, and they interpreted that oscillating velocities, pressure variations, and microstreaming created by ultrasound not only reduced the boundary layer thickness but also increased the water transfer to the air phase. In another study, ultrasound-assisted convective drying of apple shortened the drying time to 160 min, which incurred purely under convective drying [51], whereas combining the treatments of convective drying (50 °C), microwave (100 W), and ultrasound (200 W) reduced the drying time by 79%, as compared to convective drying alone for raspberries [52]. An ultrasonic pretreatment on Andean blackberry was applied before convective drying by Romero and Yépez [53], who found greater antioxidant retention than the control due to a reduced processing time and lower drying temperatures. Recently, the application of ultrasound in food-drying methods has gained popularity among researchers for the drying of hydrophilic and lipophilic nutrients for microencapsulation [54], the drying of deformable porous materials [55], and ultrasound-assisted infrared drying of jackfruit [56]. The different techniques of ultrasound-assisted drying of various fruit and vegetable crops have been explained in Table 2.

### 5.4. Ultrasound-Assisted Freezing

Freezing is a widespread method for the preservation of food products with better retention of texture, colour, taste, and nutritional value than other methods. The better quality retention is attributed to a low temperature and low water activity (aw) during the freezing operation, which results in the cryo-concentration of the liquid phase during ice crystal formation. Nucleation is the requirement for the commencement of freezing, and nucleation needs supercooling [65]. It was reported that the higher the supercooling degrees, the smaller the ice crystals occurring evenly inside or outside of the cells, and vice versa [66,67]. The location and size of the ice crystal, which depends on the freezing rate and exposure time, positively governs the quality of frozen foods. Uneven and large ice crystal formation at a slower rate of freezing is the drawback of freeze-preservation. The formation of large ice crystals would also damage food quality, including the textual attributes, nutritional value, appearance, and sensory properties. In order to overcome this problem, an accelerated rate of freezing produces small intracellular ice crystals [65]. The accomplishment of the freezing process at an optimum freezing rate in the food industries requires combinations of air freezing, immersion freezing, and cryogenic freezing [68].

**Figure 3:**
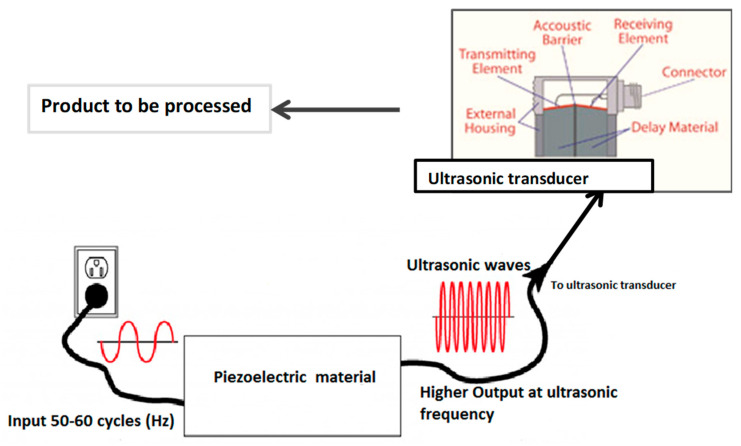
Assembly of ultrasound freezing technique.

Power ultrasonic freezing is useful in controlling the crystallization process. It plays an effective role in the initiation of nuclei and subsequent crystal growth. The arrangement of different components in ultrasonic freezing equipment has been shown in Figure 3. The main advantage of the application of power ultrasound in food freezing is that it produces evenly distributed, fine ice nucleation at an accelerated rate, thus reducing damages to the cellular structure [68]. The cavitation bubbles can act as nuclei for crystal growth, or strong forces produced by the collapse of cavitational bubbles will cause the fragmentation of already-present nuclei into smaller ones. It was reported that ultrasound can induce secondary nucleation and affect crystal growth by fracturing ice crystals [69]. Power ultrasonic waves of sound intensities higher than 1 W/cm^2^ and frequencies in the range from 20 to 100 kHz proved to be effective in the crystallization process.

The turbulence caused by micro streaming has been used to enhance the heat and mass transfer process [70]. It can also be beneficial in the freezing process, reducing both the heat and mass transfer resistance at the ice/liquid interface, resulting in an increase in the freezing rate [71]. It was reported by Xin et al. [66] that relatively higher freezing rates can be achieved at relatively low ultrasound power levels. Higher ultrasound frequencies generate greater acoustic streaming, which, in turn, increases the rate of heat transfer, which results in a shorter freezing time. The ultrasonic vibration increases the fluid-to-particle convective heat transfer coefficient and is dependent upon ultrasonic power levels [72]. The absorption of ultrasonic power by the freezing solution causes a temperature increase. The rate of the temperature rise against the time of the ultrasound application can be measured calorimetrically to calculate the actual power transferred to the freezing solution during the sonication process. The power output dissipated (P) was calculated according to Equation (3):

P = dT/dt CpM(3)

where dT/dt is the initial slope of the line representing the variation of temperature during the ultrasound exposure time, Cp is the heat capacity of the liquid medium (i.e., for water, Cp = 4.181 kJ kg^−1^ K^−1^), and M is the amount of sample treated. Then, acoustic intensity (W/cm^2^) can be obtained by the ratio of ultrasonic power to the cross-section area of the bottom of the vessel. The ultrasound processing parameters and freezing rates of some important foods are summarized in Table 3.

### 5.5. Ultrasound-Assisted Extraction

Extraction is the mass transfer process that depends on the nature of the solute and solvent and the selectivity of the solvent. The main inadequacy of conventional methods is the high-energy utilization and low extraction yield. Novel techniques, such as ultrasound-assisted extraction (UAE) and enzyme-assisted extraction (EAE) are more efficient, capable of producing high quantities of polysaccharides, and are eco-friendly, emerging as ideal alternatives to conventional techniques [75]. High-power ultrasound can be used as a tool for the extraction of essential components. A Sono-Soxhlet system consists of an ultrasonic probe immersed directly into the extraction chamber. The Sono-Soxhlet method used for lipid extracts works at an accelerated extraction rate, requiring 30 min, vs. the conventional 8 h for the production of extracts of the same quality [76].

The turbulent collapse of cavitation bubbles on the surface of the product creates micro jetting, generating surface peeling, erosion, and particle breakdown [77]. This further increases the area exposed to the ultrasonic field and facilitates the release of the compound inside the raw material into the solvent [78]. Since the mechanism of bubble formation and collapse is localised and short-lived, very little quantity of heat is transferred from the cavitational bubbles to the medium, causing only a gradual temperature increase in the medium. The cavitation produces microjets at the surface of the food material which may introduce the liquid into the solid. This effect can increase mass transfer in both directions—from the liquid to the solid, or in reverse. Therefore, cavitation-induced cell disruption and dispersion of suspended solids, coupled with enhanced mass transfer rates due to acoustic streaming, are believed to be responsible for the improved extraction [79]. The ultrasound enhanced extraction is usually carried out at a relatively low temperature. As for the bioactive and non-bioactive components, and other components which may be thermally degraded, a low temperature is beneficial to keep their bioactivity and value during extraction. Ultrasonic-Assisted Enzyme Extraction (USAE) increases the extraction yield, the rate of extraction, and reduces the time of extraction and higher throughput, along with the advantage of the usage of low temperatures and solvent volumes, which is very advantageous for the extraction of heat-liable compounds. The efficiency of the extraction process is a function of the molecular affinity between solvent and solute [80]. Some of the important ultrasound-assisted extraction processes and their effect on extraction are summarized in Table 4. The extraction with independent enzymatic hydrolysis results in greater polysaccharide yield than independent ultrasound treatments, but ultrasound-assisted enzymatic extraction results in greater yield than independent enzymatic extraction [81].

### 5.6. Ultrasonic Foaming and Defoaming

The diffusion of a gas within a liquid generated by gas bubbles separated from each other by a thin liquid film is known as liquid foam. The volume ratio of gas to liquid in this medium is extremely high, and the bulk density approaches that of the gas. If the gas phase is dissolved in the liquid phase, it can be produced by agitation and aeration of liquids. It can also be created through the evaporation of liquids, as well as through microbiological or chemical reactions that produce gases [87,88]. Cavitation bubbles generated during the sonication process were never of sufficient numbers to form foam. However, the sonication technique is used to form foam by placing the sonication horn on the air–liquid surface, by which air bubbles are entrained in the mixture as the sonication proceeds. This approach has been used to generate aerated gelatine and β-lactoglobulin gels [89]. The foaming of protein solutions can also lead to the formation of stable air-filled microcapsules. This approach has also been used as a mechanism to encapsulate volatile aromas and flavours [90]. The placement of the ultrasonic horn at a liquid–liquid interface, rather than an air–liquid interface, can lead to the formation of liquid-filled microspheres that might similarly be used to encapsulate oil-based ingredients within an aqueous food matrix.

**Figure 4:**
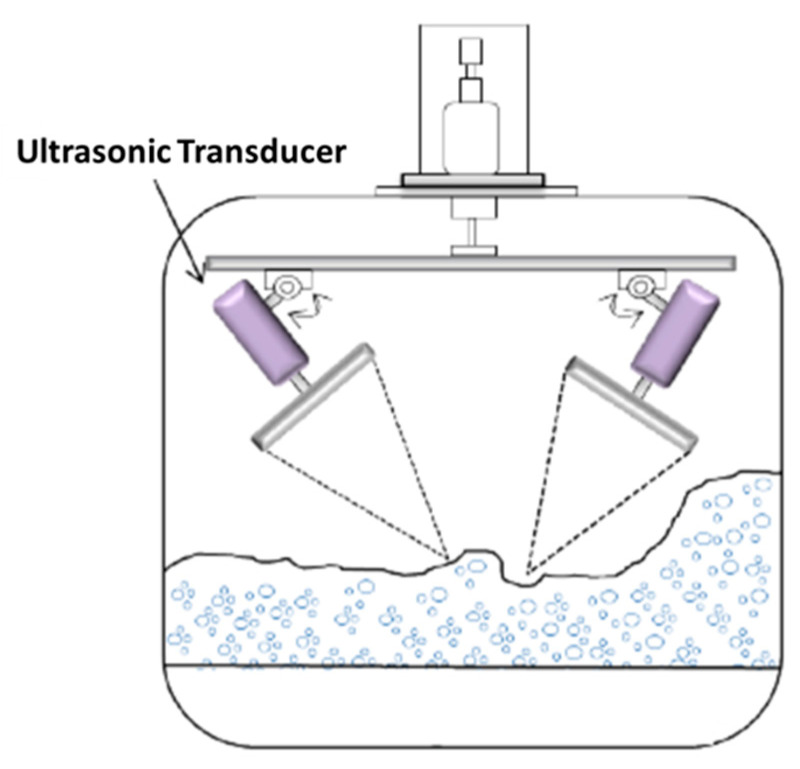
Schematic view of ultrasonic defoaming (reprinted with permission from Ref. [94] Copyright 2017 Elsevier).

Somewhat conversely, ultrasonic treatment has also been highly effective in reducing the extent of foaming [91,92]. The use of a powerful ultrasonic transducer in the air space directly above a foaming solution may produce a partial vacuum on the foam bubble surface being generated by the high acoustic pressure [92]. The schematic view of the ultrasonic defoaming equipment is described in Figure 4. The impingement of radiation pressure on the bubble surface, the resonance of the foam bubbles that create interstitial friction causing bubble coalescence, cavitation, atomizing from the liquid film surface, and/or acoustic streaming is effective in destroying the foam [92]. As a defoaming tool, Rodriguez et al. [93] have used airborne ultrasonic plate transducers. At equivalent foam concentrations, defoaming efficiency increased with increasing ultrasonic intensity (to 5 cm^3^ at 10 W/cm^2^) and treatment time. The researchers also tested how effectively ultrasonic therapy worked on two types of foam: a stable foam with a 2.85-g/L surfactant concentration and bubble sizes ranging from 0.2 to 2.0 mm, and a dry and light foam with a 0.71-g/L concentration and bubble sizes ranging from 0.2 to 10.0 mm. The liquid-to-foam volume ratios were 0.02 and 0.005, respectively. After a 98-millisecond ultrasonic treatment, the 2.85-g/L foam had a defoaming volume of 5 cm^3^, compared to 13 cm^3^ for the 0.71-g/L foam. The key benefits of airborne ultrasonic defoaming are the lack of contact with the foam, which keeps the end product sterile, ensures the ability to clear foam from flammable liquids, and ensures the lack of adjunct materials [49].

### 5.7. Functional Food Emulsions

Emulsions are a mixture of two naturally immiscible liquids that consist of minute spherical droplets of one liquid scattered in another. The functioning and stability of emulsions are governed by droplet size and polydispersity. Ultrasonic cavitation produces tremendous shear forces that can be utilised to make emulsions with very small and evenly sized droplets [95]. The oscillation and collapse of acoustic cavitation bubbles could produce a high-speed liquid jet, high temperatures, pressures, shock waves, turbulence, and intense physical shearing in the medium, among other physical phenomena [96]. The emulsification action of ultrasonography is caused by two methods. First, the sound field causes interfacial waves to form, which become unstable and cause the oil phase to disperse into the continuous water phase as mid-to-large-sized droplets. Second, cavitation’s physical processes break up these initially produced droplets of scattered oil into sub-micron-sized droplets [88]. Ultrasound frequency, power, temperature, and the surface tension or viscosity of the medium are all parameters that might affect cavitation efficiency. If the frequency is raised, for example, the power must be increased to maintain the same amount of cavitational activity. Cavitation in a liquid is more difficult to produce at higher frequencies (MHz range) because the compression and rarefaction cycles are shorter [97]. The de-emulsification method uses extremely high frequencies (MHz range). Ultrasound in the low-frequency range, particularly 20 kHz, has been proven to be effective in generating stable emulsions [98].

Li et al. [99] evaluated the energy economy and effectiveness of ultrasonic emulsification at various pressures using varied parameters (processing volume, sonication time, and amplitude) (from 20 to 120 MPa). The droplet sizes of emulsions made with various ultrasonic emulsification settings were measured. The ultrasound amplitude had a greater impact on size reduction than the ultrasonic emulsification period. Increased processing volume also resulted in smaller droplet sizes due to a decrease in energy density. Furthermore, the study found that ultrasonic devices were more energy-efficient than high-pressure homogenizers (HPH) because sonication could make nanoemulsions while using less electrical energy than HPH. Transparent emulsions of ethylene dioxythiophene (EDOT) monomer formed by sequential ultrasonic emulsification at 20 kHz, 1.6 MHz, and 2.4 MHz were recorded by [100] mainly by interfacial waves and cavitation. Stable food-based emulsions of flaxseed oil in skim milk can be formed by ultrasonic emulsification, and emulsion was stable for at least 7 days [101]. Ultrasonics can be used to spread various organic/oil phases into various aqueous phases in a regulated manner, resulting in emulsified goods that are shelf-stable and appealing to the eye.

## 6. Pros and Cons of Using Ultrasonication

In the food industry, ultrasound applications provide various benefits:

- Mechanical effects generated by ultrasound, turbulence, shock waves, microstreaming, etc., increase mass transfer within the medium that may positively influence chemical reactions and other processes. Ultrasound waves are non-toxic, safe, and environmentally benign. Ultrasonication is a microbial inactivation approach that can be used with a variety of thermal and non-thermal treatments;
- Ultrasound has the ability to start reactions without the use of external chemicals. External reagents are not required because the collapsing bubbles generate radicals that can perform redox reactions. The products produced without external chemical reagents would be purer, and the processing atmosphere would be ‘greener’;
- Processes, such as emulsion polymerisation and microsphere creation, necessitate both physical and chemical effects, which sonication can provide. In addition to the substantially faster reaction rates and higher monomer-to-polymer conversion rates, ultrasonic polymerization has been proven to produce polymer particles with a high molecular weight;
- When compared to other juice-extraction processes, ultrasound will be more efficient in increasing juice production, cutting processing time by 55% and temperature by 16%;
- During processing, ultrasonic-treated items will lose the least amount of flavour, colour, and other nutritional ingredients.

Despite the many benefits of employing ultrasound in preservation, processing, extraction, emulsification, and centrifugation homogenization, there are some limitations, including:

- Their inability to obtain large-scale ultrasonic reactors is a problem. Ultrasonic equipment is now custom-made for individual uses, which raises the expense of implementing this technology in businesses;
- In some applications, direct contact between processing liquids and ultrasonic horns is a problem. This could be avoided by using a flow-through ultrasonic reactor with short residence durations and creating non-contact reactors;
- The free radicals produced through cavitation could impact the product and harm the consumer.

## 7. Health Effects of Ultrasound

Undesirable subjective effects, such as fullness in the ear, headaches, and malaise, were experienced for subjects exposed to ultrasonic energy (frequencies > 17 kHz and sound level > 70 dB). Scientists have also reported that airborne ultrasound has the potential to cause nausea and fatigue. Skin temperature could slightly increase when the body is subjected to sound pressure levels of 140–150 dB at ultrasonic frequencies, whereas further increases for sound doses above 180 dB would be lethal to humans. Mawson et al. [102] analysed the formation of metal particulates from ultrasonic transducers and found no result in support of the formation of harmful nanoparticles (<80 nm). However, the National Occupational Health and Safety Commission reported that there is less substantiation for the specific unfavourable effects of ultrasound [103].

## 8. Conclusions

Ultrasonication is considered a clean technology as compared to other physical and chemical food processing operations, developing a high potential for consumer acceptance. As a result of these acoustic effects, power ultrasound has shown to be an excellent technique for initiating ice crystal nucleation, controlling the size and shape of ice crystals, and accelerating the formation of ice crystals, improving the pace of freezing and the quality of frozen goods. The use of ultrasound can have a strong presence in food industries if its potential for the development of new products is fully evolved. Though this technology has been successfully used for extraction, pasteurization, cutting, foaming, drying, to mention a few, the economic feasibility of using this technology for preservation needs to be examined as well. Although the use of power ultrasound in the food industry has been generating vast applications, there is still a need to generate more systematic data about the responses of microorganisms, food enzymes, and food components (proteins, carbohydrates, lipids, nutrients, plant and animal cells, etc.) to ultrasound treatment. Additionally, the microbial and enzyme inactivation mechanisms and kinetics are still at large. The factors that affect the cavitation intensity and the methods needed to quantify the cavitation activity need to be studied. More emphasis should be laid on the development of food-grade ultrasound treatments systems so that more data on the effect of ultrasound on food quality is generated. While this technology has great promises, it will have to be carefully developed and scaled up for every single food application.

## Figures and Tables

**Table 1:** D values of microorganisms inactivated by using heat, ultrasound, thermosonication, monosonication, and manothermosonication.

Temperature (°C)	Organism	D Value (min)				Reference
		Heat	Ultrasound	Thermosonication	Monosonication/Manothermosonication (MTS)	
60	*Saccharomyces cerevisiae*	3.53	3.1(20 kHz, 124 μm)	1.9 (1 min US, 55 °C) 0.73 (1 min US, 60 °C)	-	[36]
61	*Escherichia coli K*12	0.79	1.01	0.44 (100 kPa)	0.40 (300 kPa)/0.27 (MTS 300 kPa 61 °C)	[37]
56	*Cronabacter sakazakii*	0.86	-	-	0.28 (MTS-20 kHz, 117 μm, 200 kPa, 56 °C)	[38]
55	*Aspergillus flavus*	17.40	-	5.06 (120 μm) 4.94 (120 μm, 500 ppm vanillin (Vi)) 1.09 (120 μm, 500 ppm potassium sorbate (KS))		[39]
60		2.60	-	1.20 (120 μm) < 0.5 (120 μm, 500 ppm Vi) < 0.5 (120 μm, 500 ppm KS)		
50	*Penicillium digitatum*	25.42	-	9.59 (120 μm) 8.57 (120 μm, 500 ppm Vi) 7.15 (120 μm, 500 ppm KS)		[39]
52.5		13.30	-	5.33 (120 μm) 6.47 (120 μm, 500 ppm Vi) 4.19 (120 μm, 500 ppm KS)		
63	*Listeria innocua*	30	-	10 (400 W, 24 kHz, 120 μm)		[40]
Ambient	Mesophilic aerobic, Lactic acid bacteria, Coliform bacteria, yeast		750 W, 20 kHz, 6.8–126 μm			[28]
25	*Salmonella Typhimurium, Escherichia coli*		80 and 37 kHz, (330 W), pulsed modes, frequency amplitude (40% and 100%)			[41]

**Table 2:** Effect of ultrasound during drying.

Drying Technique	Ultrasound Processing Parameters	Sample	Inference	Reference
Ultrasound-assisted osmotic drying	Indirect sonication: 25 kHz, 1.75 kW Osmotic solution: 70° Brix Immersion time: 60 min Air drying: 70 °C	Guava slices	Initial moisture content: 91.3 ± 0.6% wet basis (w.b.) Final moisture content: 19.5 ± 3 (w.b.) Total dehydration time: 300 min. Drying time reduced by 33%.	[57]
Ultrasound-assisted convective drying	Ultrasound: 21.8 kHz, 60 W Air drying: 70 °C	Strawberry	Initial moisture content: 90.5 ± 0.27 (w.b.) Final moisture content: 23.07% (w.b.). Total drying time: 2.2 h. Drying time reduced by 44%.	[58]
Ultrasound-assisted osmotic dehydration	Ultrasound: 25 kHz, 700 W	Cherries	It was proved that intermittent drying of cherries preceded by ultrasonic-assisted osmotic dehydration contributes to shorter drying time, better colour preservation, and smaller water activity.	[59]
Ultrasound-assisted convective drying	Ultrasound: 21.8 kHz, 30.8 W Air drying: 70 °C	Passion fruit peel	Initial moisture content: 87.5± 1.9 (w.b.) Final moisture content: 32% (w.b.) Total drying time: 3.9 ± 0.7 h.	[60]
Ultrasound-assisted radiation drying	Ultrasound: 1200 W, 20 kHz Sonication time: 5 s Drying: 62 °C	Carrot slices	Final moisture content: 10 ± 0.5% (d.b.) Drying time increased with increasing ultrasonic power levels.	[61]
Ultrasound-assisted vacuum drying	Sonication time: 10 s Drying: 65–75 °C	Carrot slices	Final moisture content: 12–13% dry basis (d.b) Drying time was decreased by 53%.	[62]
Ultrasound-assisted heating	1000 W and 50 ℃	Ham slices	Decrease of 0.65-fold in adhesiveness values. Population of free water increased from 2.71% to 11.35%. Decreased the content of rancid and sour compounds. Accelerated the formation of esters.	[63]
Ultrasound-assisted microwave dryer	28 kHz, 70 W, 30 min	Carrot slices	Reduction in drying time by 63%. Least-specific energy consumption: 23.75 ±2.22 MJ/kg Lowest shrinkage: 31.8 ±1.1%	[64]

**Table 3:** Effect of ultrasound during freezing.

Processing Parameter	Nucleation Temperature Range	Sample	Inference	Reference
Ultrasound: −0.1 to −2.1 °C Ultrasound Intensity: 0.09 to 0.51 W/cm^2^ Sonication: 30 s	−3.43 to −2.36 °C −3.43 to −2.38 °C	Strawberry	Nucleation temperature increased with increase of US temperature. No linear relationship between USI and the degree of super cooling. Shortest CFT observed at 0.51 W/cm^2^.	[73]
Ultrasound: −0.5 to −2.0 °C Ultrasound Intensity: 0.09–0.37 W/cm^2^ Sonication: 3–15 s	−1.6 to −2.75 °C	Radish	Nucleation temperature increased with increase of US temperature. Increasing USI, NT increases first and then decreases with increasing US duration. US 0.226 W/cm^2^ at −0.5 °C for 7 s is enough for commencement of nucleation.	[74]
Ultrasound: 125–190 W; 20–30 kHz Sonication: 60 s	-	Broccoli	Higher freezing rate can be achieved at relatively low-power ultrasound. Shorter freezing times were observed at relatively lower ultrasound frequency. At relatively low power levels (125 W), the freezing time was found to be significantly shorter at 30 kHz than at 20 kHz, whereas at relatively high power levels (175 W), the freezing time at 30 kHz was significantly longer than at 20 kHz.	[66]
Ultrasound: −30 °C; 90–630 W; 45 kHz	−2 to −4 °C	Penaeus chinensis	Optimized ultrasonic parameters were power 191.97 W, 4.92 s on/3 s off. Optimized ultrasonic parameters provide an economic and effective way for obtaining high-quality frozen Penaeus chinensis.	[72]

**Table 4:** Effect of ultrasound on extraction.

Ultrasound Process Parameter	Extract	Solvent	Sample	Inference	Reference
Ultrasound: 100–250 W Temperature: 45–60 °C Liquid-to-Solid Ratio (LSR): 30–50 mL/g Time: 20–40 min.	Melanin	NaOH	*Auricularia Auricula*	Increasing US power, temperature, LSR, and duration may enhance the melanin yield. Optimum extraction conditions: (120.05 mg/g)−63 °C, 43 mL/g, 36 min, two extractions.	[82]
Ultrasound: 80–120 W LSR: 20:1–40:1 mL/g Temperature: 40–60 °C Time: 30–50 min.	Polysaccharide	Distilled water	*Nephelium Lappaceum L*.	Polysaccharide yield increases with increasing ultrasound power (USP 80–100 W, temperature: 40–50 °C, duration up to 40 min, LSR 20:1–30:1 mL/g), respectively, and then decreases accordingly. Optimum extraction conditions: (8.32%)–53 °C, 110 W, 41 min, 32:1 mL/g.	[83]
Ultrasound: 40–120 W; 40 kHz Temperature: 40–80 °C Enzyme concentration: 0.5 to 2.5% Time: 10–80 min.	Polysaccharides	Cellulose	*Lycium barbarum*	Polysaccharide yield increases with increasing (USP 40–80 W, temperature: 40–60 °C, duration up to 20 min, Cellulose concentration: 0.5% to 2.0%), respectively, and then decreases accordingly. Optimum extraction conditions: (6.32%)–55.79 °C, 78.6 W, Cellulose concentration: 2.15%, 20.29 min.	[84]
	Fructo-oligosaccharides (1-kestone), Nystone, 1F-*β*-fructofuranosylnystose; Raffinose family oligosaccharides (Raffinose and Stachyose)	Ethanol, Methanol and Acetone	Fruits (Blueberry, Nectarine, Raspberry, Watermelon) Vegetables (Garlic, Jerusalem Artichoke, Leek, Scallion, Spring Garlic, White Onion)	USAE was an efficient method for the extraction of oligosaccharides at extraction time of 10 min, temperature: 40 °C, and ethanol concentration 63% *v*/*v*. Aqueous acetone was slightly better than aqueous methanol and aqueous ethanol in USAE of oligosaccharides under the same extraction conditions (50% *v*/*v*, 50 °C, and extraction: 10 min). Extraction of the oligosaccharides increased with increase of the ethanol concentration up to 60%. The concentration of the extracted oligosaccharide increased when the extraction temperature increased from 20 °C to 50 °C. The highest increase of total oligosaccharide was spotted in the Jerusalem artichoke (1.96% conventional extractions) to (7.17% USAE).	[85]
Ultrasound: 37 kHz;Temperature: 60–80 °C Sonication time: 15 min	Pectin	Ammonium oxalate/oxalic acid	Tomato waste	At 60 °C, the pectin yield obtained was higher for the conventional extraction, which obtained a higher yield than ultrasound-assisted extraction. At 80 °C, the pectin yield obtained was comparable for both the methods, but better quality of pectin was obtained with ultrasound-assisted extraction.	[86]
